# Favipiravir Versus Arbidol for Clinical Recovery Rate in Moderate and Severe Adult COVID-19 Patients: A Prospective, Multicenter, Open-Label, Randomized Controlled Clinical Trial

**DOI:** 10.3389/fphar.2021.683296

**Published:** 2021-09-02

**Authors:** Chang Chen, Yi Zhang, Jianying Huang, Ping Yin, Zhenshun Cheng, Jianyuan Wu, Song Chen, Yongxi Zhang, Bo Chen, Mengxin Lu, Yongwen Luo, Lingao Ju, Jingyi Zhang, Xinghuan Wang

**Affiliations:** ^1^ Clinical Trial Center, Zhongnan Hospital of Wuhan University, Wuhan, China; ^2^ Department of Anesthesiology, Zhongnan Hospital of Wuhan University, Wuhan, China; ^3^ Center for Life Sciences, Peking University, Beijing, China; ^4^ Euler Technology, Beijing, China; ^5^ Wuhan Leishenshan Hospital, Wuhan, China; ^6^ Department of Epidemiology and Biostatistics, School of Public Health, Tongji Medical College, Huazhong University of Science and Technology, Wuhan, China; ^7^ Department of Respiratory Medicine, Zhongnan Hospital of Wuhan University, Wuhan, China; ^8^ Department of Urology, Zhongnan Hospital of Wuhan University, Wuhan, China; ^9^ Department of Infectious Diseases, Zhongnan Hospital of Wuhan University, Wuhan, China; ^10^ Department of Cardiology, The Third People’s Hospital of Hubei Province, Wuhan, China; ^11^ Center for Evidence-Based and Translational Medicine, Zhongnan Hospital of Wuhan University, Wuhan, China

**Keywords:** COVID-19, SARS-CoV-2, favipiravir, umifenovir (arbidol), randomized controlled trial

## Abstract

**Background:** In addition to supportive therapy, antiviral therapy is an effective treatment for coronavirus disease 2019 (COVID-19).

**Objective:** To compare the efficacy and safety of favipiravir and umifenovir (Arbidol) to treat COVID-19 patients.

**Methods:** We conducted a prospective, randomized, controlled, open-label multicenter trial involving adult patients with COVID-19. Enrolled patients with initial symptoms within 12 days were randomly assigned in a 1:1 ratio to receive conventional therapy plus Arbidol (200 mg*3/day) or favipiravir (1600 mg*2/first day followed by 600 mg*2/day) for 7 days. The primary outcome was the clinical recovery rate at day 7 of drug administration (relief for pyrexia and cough, respiratory frequency ≤24 times/min; oxygen saturation ≥98%). Latency to relief for pyrexia and cough and the rate of auxiliary oxygen therapy (AOT) or noninvasive mechanical ventilation (NMV)/mechanical ventilation (MV) were the secondary outcomes. Safety data were collected for 17 days.

**Results:** A total of 240 enrolled COVID-19 patients underwent randomization; 120 patients were assigned to receive favipiravir (116 assessed), and 120 patients were assigned to receive Arbidol (120 assessed). The clinical recovery rate at day 7 of drug administration did not significantly differ between the favipiravir group (71/116) and Arbidol group (62/120) (*p* = 0.1396, difference in recovery rate: 0.0954; 95% CI: −0.0305∼0.2213). Favipiravir contributed to relief for both pyrexia (difference: 1.70 days, *p* < 0.0001) and cough (difference: 1.75 days, *p* < 0.0001). No difference was observed in the AOT or NMV/MV rate (both *p* > 0.05). The most frequently observed favipiravir-associated adverse event was increased serum uric acid (16/116, OR: 5.52, *p* = 0.0014).

**Conclusion:** Among patients with COVID-19, favipiravir, compared to Arbidol, did not significantly improve the clinical recovery rate at day 7. Favipiravir significantly improved the latency to relieve pyrexia and cough. Adverse effects caused by favipiravir are mild and manageable.

**Clinical Trial Registration:** Clinical trial registered with Chictr.org.cn, (ChiCTR2000030254).

## Introduction

Coronavirus disease 2019 (COVID-19), caused by SARS-CoV-2, was first discovered in Dec. 2019. As of Mar. 20, 2021, the WHO reported 121,759,109 confirmed cases across more than 200 countries with a global mortality rate of 2.21% (Roser et al., 2020; oronavirus disease, 2020).

In the early phase of the COVID-19 outbreak in Wuhan (January 2020-February 2020), no validated treatment existed for COVID-19. The main strategies were symptomatic and supportive care, such as maintaining vital signs, maintaining oxygen saturation and blood pressure, and treating complications, such as secondary infections or organ failure. The standard practice of care focuses on treating the clinical symptoms, including pyrexia, cough, and acute respiratory distress syndrome (ARDS), of patients with supportive care, such as fluid management and auxiliary oxygen therapy. No proven clinical efficacy of antiviral agents for COVID-19 has been reported, while some (Remdesivir, hIFNα-2b, Ribavirin, Chloroquine and Arbidol) are currently under clinical trials for COVID-19 (Dong et al., 2020; Wang et al., 2020).

The SARS-CoV-2 and influenza viruses exhibit similar clinical features with similar organ tropism. Because both viruses are RNA viruses depending on RNA-dependent RNA polymerase (RdRp) to replicate, the RdRp inhibitor Arbidol (brand name for umifenovir), approved for influenza in Russia and China, has been proposed as a standard care option for COVID-19, mainly based on its mechanism of action (MoA) and its effects in treating influenza-associated pneumonia (Shi et al., 2007; Hulseberg et al., 2019; Pshenichnaya et al., 2019). Additionally, Arbidol treatment for COVID-19, including five retrospective cohort studies, two prospective cohort studies, and one RCT, suggested that Arbidol could accelerate and enhance the process of viral clearance (Nojomi et al., 2020; Zhang et al., 2020). A retrospective cohort study in China suggested that Arbidol could accelerate and enhance the process of viral clearance, improve focal absorption on radiologic images, and reduce the demand for HFNC oxygen therapy in hospitalization (Xu et al., 2019).

Favipiravir, an antiviral drug targeting RdRP-halting viral replication (Goldhill et al., 2018), approved in Japan for influenza, has an IC50 of 0.013–0.48 μg/ml for influenza A. Compare this finding with the IC50 of 2.7–13.8 μg/ml of Arbidol (Furuta et al., 2002). Arbidol is an antiviral agent with a unique mechanism of action targeting the S protein/ACE2 interaction and inhibiting membrane fusion of the viral envelope. Therefore, we consider that favipiravir might serve as a potential candidate that is superior to Arbidol for COVID-19. The effectiveness of Arbidol in the treatment of influenza and a rapid reduction in the main clinical symptoms in adult patients was demonstrated (Leneva et al., 2016; Leneva et al., 2019). Specifically, we hypothesized that favipiravir would be superior to Arbidol to improve the clinical recovery rate at day 7 of drug administration and alleviate the clinical symptoms, namely, improvement in pyrexia, cough and oxygenation. To evaluate the clinical efficacy and safety of favipiravir versus Arbidol as a treatment for COVID-19, we conducted a prospective, randomized, controlled, open-label multicenter trial in adult patients with COVID-19.

## Methods

### Patient Description

All enrolled patients were inpatients. Patients were assessed for eligibility based on (Roser et al., 2020) age 18 years or older (oronavirus disease, 2020), voluntary informed consent (Dong et al., 2020), initial symptoms within 12 days, and (Wang et al., 2020) diagnosis of COVID-19 pneumonia.

Hence, male and female adult patients with clinically confirmed COVID-19, including moderate, severe or critical types of COVID-19, were eligible. All first-listed diagnoses of hospitalized COVID-19 patients presented positive qRT-PCR on admission, according to the Chinese Diagnosis and Treatment Protocol for Novel Coronavirus Pneumonia from the sixth and seventh editions (Feb. 18, 2020-Mar. 4, 2020) (National Health Commission and State Administration of Traditional Chinese Medicine, 2020a; National Health Commission and State Administration of Traditional Chinese Medicine, 2020b). To confirm that enrolled patients were still in the active disease stage, we performed qRT-PCR for the patients at the time of enrollment only to establish the baseline level, together with laboratory biochemistry and chest CT scans. Characteristic findings on CT imaging include multiple, patchy, ground-glass opacities followed by subpleural linear abnormalities, crazy-paving patterns, and consolidation shadows, mainly distributed in the peripheral and subpleural areas of both lungs. Patients were excluded if they met any of the following criteria (Roser et al., 2020): allergic to favipiravir or Arbidol (oronavirus disease, 2020), elevated ALT/AST (>6x upper limit of normal range) or chronic liver disease (cirrhosis at grade Child-Pugh C) (Dong et al., 2020), severe/critical patients whose expected survival time was <48 h (Wang et al., 2020), female in pregnancy (Pshenichnaya et al., 2019), HIV infection, or (Hulseberg et al., 2019) considered unsuitable by researchers for the patient’s best interest. Written informed consent was obtained from all patients or their authorized representatives if the patient was unable to write physically.

### Study Design

**FIGURE 1:**
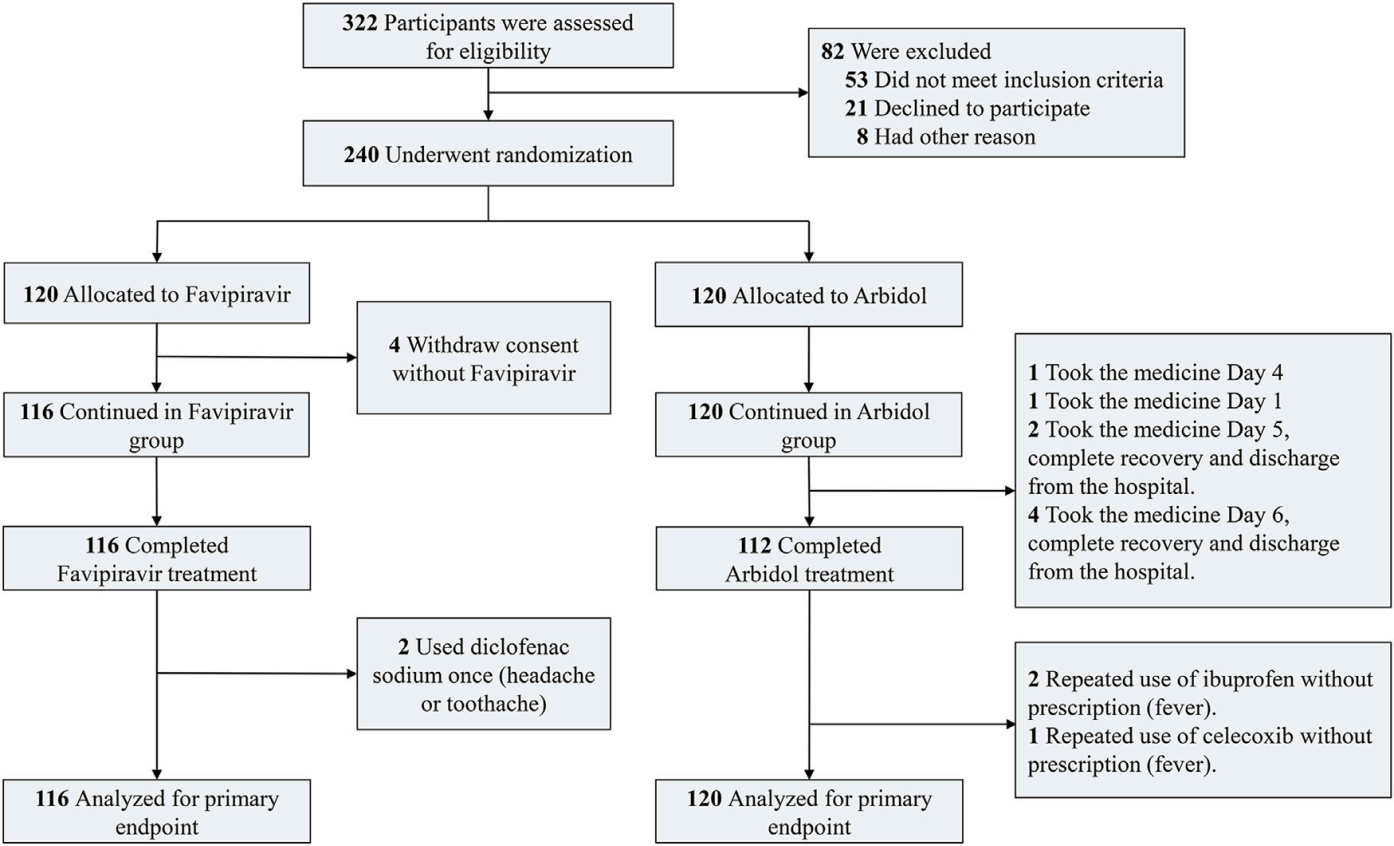
Flow diagram of the study.

The study was designed as a prospective, randomized, controlled, open-label multicenter trial (Figure 1) conducted from Feb. 20 to Mar. 1, 2020 in three hospitals [Zhonghan Hospital of Wuhan University (ZNWU); Leishenshan Hospital (LSS); and the Third Hospital of Hubei Province (HBTH)] of Wuhan, Hubei, China. A total of 240 patients with COVID-19 pneumonia were recruited from the three hospitals (120 from ZNWU, 88 from LSS, and 32 from HBTH). All participants received either favipiravir (1600 mg/600 mg; first/follow-up doses daily, BID) or Arbidol (200 mg, three times daily) plus standard care for 7 days. Because of the emergency nature of this matter, we did not prepare visually similar (placebo) pills of Arbidol for favipiravir. The treatment could be extended to 10 days at the judgment of the investigator for the patient’s best benefit. Standard care could comprise, as for the patient’s best interest, traditional Chinese herbal medicine, antibiotics, additional antiviral treatment, immunomodulatory drugs, steroids, psychotic drugs, nutrition support, cardiovascular drugs, supportive oxygen, noninvasive positive pressure ventilation (NPPV) or invasive ventilation. Randomized open labeling (1:1 ratio between Arbidol and favipiravir) was produced by the professional statistical software SAS (version 9.4) and assigned to patients. All enrolled patients included moderate cases (fever and respiratory symptoms with radiological findings of pneumonia) and severe cases. Severe cases met any of the following criteria (Roser et al., 2020): respiratory distress (≥30 breaths/min) (oronavirus disease, 2020), oxygen saturation ≤93% at rest, and (Dong et al., 2020) arterial partial pressure of oxygen (PaO_2_)/fraction of inspired oxygen (FiO_2_) ≤300 mmHg (1 mmHg = 0.133 kPa). Cases with chest imaging that showed obvious lesion progression within 24–48 h > 50% were managed as severe cases. The study was approved by the Institutional Ethics Committee (No. 2020040) (additional details in Protocol and SAP). On-site study monitoring and data management were performed by a third-party CRO (Shoufu Medical Research Organization, Beijing).

### Measurements

Patients were assessed at the time of enrollment for basic physical parameters, body temperature, chronic viral co-infections, HCG (for females of childbearing age), COVID-19 clinical classification, SpO_2_, chest CT, IL-6, blood biochemistry, urinalysis, coagulation function, C-reactive protein and SARS-CoV-2 nucleic acid (additional details in Protocol and SAP). The clinical classification of moderate, severe and critical COVID-19 patients was performed according to the Chinese guidelines (Furuta et al., 2002). After enrollment, blood biochemistry, urinalysis, coagulation function, C-reactive protein and SARS-CoV-2 nucleic acid were examined on the third (D3 ± 1 day) and seventh days (D7 ± 2 days), with an additional chest CT on the seventh day. The axillary temperature, respiratory rate, oxygen saturation without supportive oxygen, and usage of auxiliary oxygen therapy (AOT)/noninvasive mechanical ventilation (NMV)/mechanical ventilation (MV) were recorded during the daily follow-up visit. Repeated measurements were made at least twice at each follow-up visit. Resting body temperature and oxygen saturations were measured every morning and afternoon by the nursing staff. The measurements were taken after 15 min of rest at room temperature (23 ± 2°C). The cough was collected from the log card, which the subjects filled out once a day. The severity of cough was defined as follows: 0-no cough at all; 1-occasional, mild cough; 2-moderate, paroxysmal cough; and 3-severe, strenuous cough. Mild or no cough is defined as cough relief. Adverse events and concomitant medication were observed.

### Outcome Definitions

The primary outcome was the clinical recovery rate at 7 days of drug administration. Clinical recovery was defined as continuous (>72 h) recovery of body temperature, respiratory rate, oxygen saturation and cough relief after treatment, with the following quantitative criteria: axillary temperature ≤36.6°C, respiratory frequency ≤24 times/min, oxygen saturation ≥98% without oxygen inhalation, and mild or no cough. Secondary outcomes included the latency to pyrexia relief (for patients with pyrexia at the time of enrollment); the latency to cough relief (for patients with moderate or severe cough at the time of enrollment); the rate of AOT or NMV/MV, all-cause mortality, dyspnea, rate of respiratory failure (defined as SpO_2_ ≤90% without oxygen inhalation or PaO_2_/FiO_2_ <300 mmHg, requires oxygen therapy or additional respiratory support); and the rate of patients who needed to receive intensive care in the ICU. Safety outcomes included adverse events that occurred during treatment and premature discontinuation.

### Statistical Analysis

With limited knowledge of the efficacy of Arbidol, we assumed a 50% clinical recovery rate at day 7 of drug administration for the Arbidol group. The superior clinical efficacy of favipiravir was then expected to be at least 70%. With α = 0.025 (single side), β = 0.20, and power = 0.80, we estimated that 92 participants were required for each group. The sample size increased by approximately 20%, considering shedding/elimination. Hence, the trial was designed to include 240 participants in the group, including 120 in the experimental group and 120 in the control group. SAS (v9.4) software was used for statistical analysis. For the primary outcome, the comparison between the experimental group and the control group adopted the optimal test. The bilateral 95% CI of the difference between the clinical recovery rate of the experimental group and the control group was calculated. If the lower limit was larger than 0, it was considered that the experimental group was superior to the control group. The log rank test was used to compare the recovery latency between the two groups. For secondary outcomes, the Student’s *t*-test or Wilcoxon rank sum test (if the *t*-test was not applicable) was performed for safety indicators and continuous variables, and the Wilcoxon rank sum test was used for grade variables. Frequency or composition (%) was used for the statistical description of categorical variables, and the chi-square test or Fisher’s exact test was used for comparisons between groups. For all statistical tests, *p* < 0.05 (bilateral) was considered statistically significant.

### The Details of Ancillary Drugs

Details of ancillary drug use are listed in Supplementary Table S2 and Supplementary Figure S3. The most frequently used ancillary treatments were as follows: Moxifloxacin Hydrochloride tablets (22/18, favipiravir/Arbidol group); Cephalosporins (11/5); Antiviral drugs, other than the experimental drugs (11/27); Glucocorticoid (5/10); and Human Serum Albumin (4/5). In addition to the abovementioned medicines, the following Chinese herbal medicines were widely used among the patients: Lianhua Qingwen capsule (a prespecified Chinese herbal medicine recipe for respiratory contagious diseases, 23/30); Qiangli Pipa Lu (a syrup for cough relief, 12/15); and Xuebijing injection (a prespecified Chinese herbal medicine recipe for anti-inflammation, 8/10). We found that while additional antiviral treatments were more frequent in the Arbidol group (*p* = 0.0045), they did not positively contribute to the clinical recovery rate at day 7 of drug administration. Ancillary treatments differ between moderate and severe/critical patients.

## Results

### Clinical Characteristics of Patients

A total of 240 patients with COVID-19 were enrolled, of whom 236 took at least one dosage of the drug and were considered as the full analysis set (FAS) (Supplementary Figures S1, S2). The FAS set included 116 patients in the favipiravir group and 120 in the Arbidol group (Table 1). In the favipiravir group, 59 (50.86%) were males, 57 (49.14%) were females, 87 (75.00%) were <65 years, 29 (25.00%) were ≥65 years, 36 (31.03%) had hypertension and 14 (12.07%) had diabetes. In the Arbidol group, 51 (42.50%) were males, 69 (57.50%) were females, 79 (65.83%) were <65 years, 41 (34.17%) were ≥65 years, 30 (25.00%) had hypertension, and 13 (10.83%) had diabetes.

**TABLE 1:** Basic characteristics of the participants.

Variables	Favipiravir group (*n* = 116)	Arbidol group (*n* = 120)	*p* value
Gender, *n* (%)			0.2473
Female	57 (49.14)	69 (57.50)	
Male	59 (50.86)	51 (42.50)	
Age (years), *n* (%)			0.1232
<65	87 (75.00)	79 (65.83)	
≥65	29 (25.00)	41 (34.17)	
Time from symptom onset to starting study treatment, days	9 (7–12)	9 (6–12)	0.6941
Early (≤ 10 days from symptom onset)	84/116 (72.4%)	89/120 (74.1%)	0.7609
Late (> 10 days from symptom onset)	32/116 (27.5%)	31/120 (25.8%)	
Clinical Classification, *n* (%)			0.1540
Moderate	98 (84.48)	111 (92.50)	
Severe	16 (13.79)	8 (6.67)	
Critical	2 (1.72)	1 (0.83)	
Hypertension, *n* (%)	36 (31.03)	30 (25.00)	0.3018
Diabetes, *n* (%)	14 (12.07)	13 (10.83)	0.7656
Insomnia, *n* (%)	16 (13.79)	29 (24.17)	0.0426
Conjunctivitis, *n* (%)	6 (5.17)	7 (5.83)	1.0000^a^
Oxygen saturation, %			
Mean (SD)	93.50 (3.05)	94.11 (1.93)	0.0681
Min-Max	78–96	82–96	
Signs and symptoms, *n* (%)			
Pyrexia	64 (55.17)	61 (50.83)	0.5911
Fatigue	40 (34.48)	27 (22.50)	0.0579
Dry cough	70 (60.34)	64 (53.33)	0.3393
Myalgia	2 (1.72)	3 (2.50)	1.0000^a^
Dyspnea	9 (7.76)	4 (3.33)	0.2285
Expectoration	13 (11.21)	11 (9.17)	0.7619
Sore throat	9 (7.76)	17 (14.17)	0.1726
Diarrhea	22 (18.97)	15 (12.50)	0.2354
Dizziness	1 (0.86)	5 (4.17)	0.2306
Laboratory findings			
Nucleic acid tests, *n* (%)	*n* = 116	*n* = 120	0.4202
Positive	54 (46.55)	46 (38.33)	
Suspected	6 (5.17)	6 (5.00)	
Lymphocyte count, ×10^9^/L	*n* = 116	*n* = 120	0.5316
Mean (SD)	0.95 (0.25)	0.97 (0.34)	
Min-Max	0.54–2.14	0.36–2.21	
Erythrocyte sedimentation rate (ESR)	*n* = 114	*n* = 120	0.9498
Mean (SD)	17.24 (14.34)	17.34 (10.76)	
Min-Max	2.00–96.00	2.00–61.00	
C-reactive protein (CRP)	*n* = 116	*n* = 118	0.4796
Mean (SD)	10.91 (21.55)	9.19 (14.92)	
Min-Max	0.50–212.60	0.50–111.90	
Chest CT (*n* = 235 with data), *n* (%)	116	119	0.7635
COVID-19 pneumonia	112 (96.55)	114 (95.80)	

a *t*-test was performed for continuous variables, frequency or composition (%) were used for statistical description of classification indexes, and Chi-square test or Fisher’s exact test was used for comparison between groups.

The main signs and symptoms for enrolled patients were pyrexia, fatigue, dry cough, myalgia, dyspnea, expectoration, sore throat, diarrhea, dizziness, insomnia and conjunctivitis, none of which were significantly different between the groups. There was no difference between the time from the onset of patient symptoms to the time of treatment initiation between the groups. Patients who were symptomatic for 10 days or less at the time of enrollment received favipiravir (72.4%) or Arbidol (74.1%) treatment (Table 1). Neither the SARS-CoV-2 nucleic acid test positive rate, lymphocyte count, erythrocyte sedimentation rate nor the C-reactive protein differed between the groups (Table 1). In addition, 116 patients in the favipiravir group and 119 in the Arbidol group underwent a chest CT, of whom 112 (96.55%) and 114 (95.80%) were diagnosed with COVID-19 pneumonia, according to the diagnostic criteria (*p* = 0.7635, *t*-test). Overall, no significant difference in basic characteristics of patients between the two groups was observed. However, we noticed a marginally increased ratio of severe to critical patients in the favipiravir group (16 (severe) + 2 (critical); SpO_2_ 93.508) compared to the Arbidol group (8 + 1; SpO_2_ 94.116) (*p* = 0.0658, Fisher’s exact test, OR: 2.25 [0.91–5.98]; *p* = 0.0681).

### Comparison of the Clinical Recovery Rate at Day 7 of Favipiravir and Arbidol in COVID-19 Patients

**FIGURE 2:**
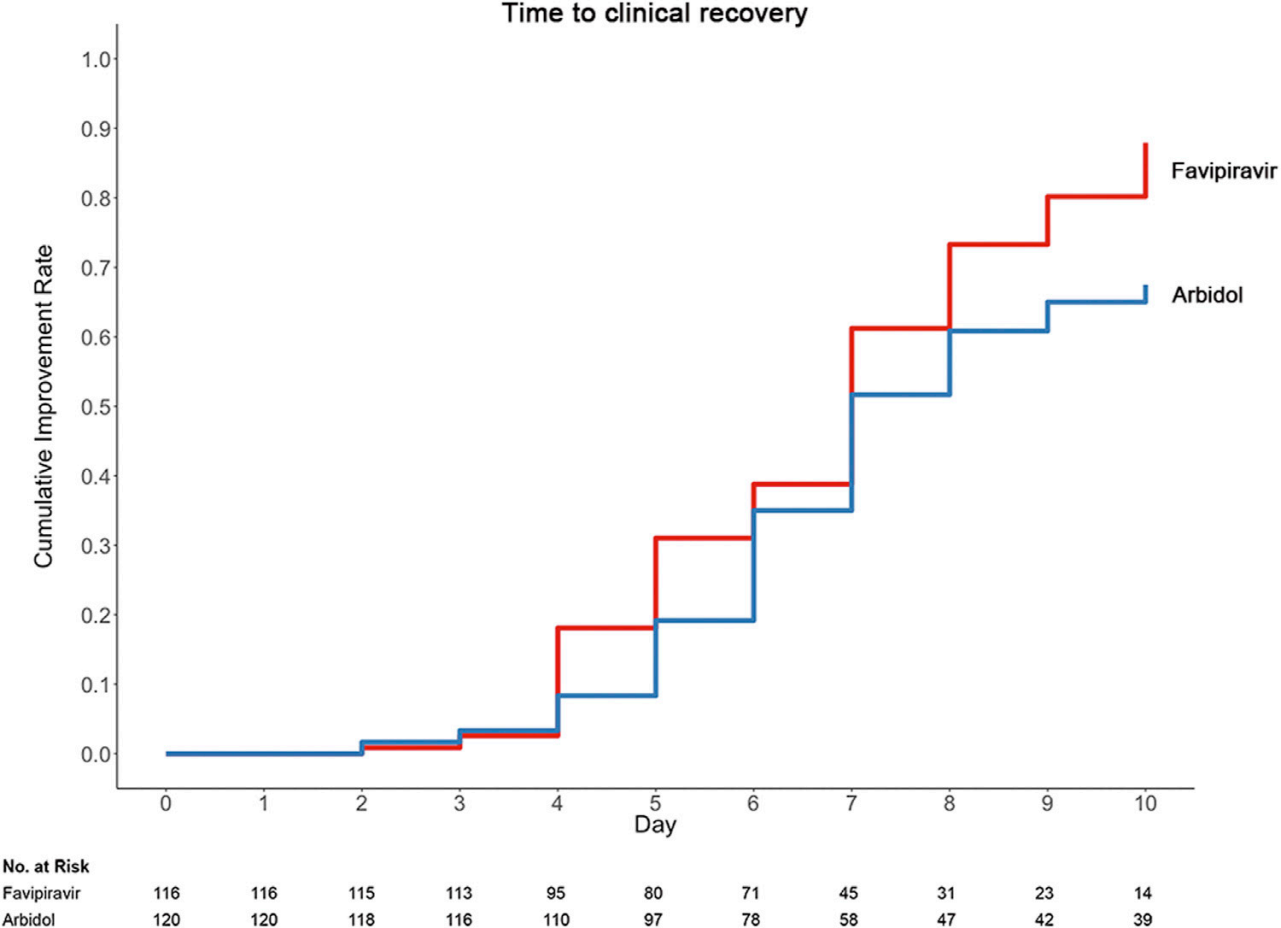
Time to clinical recovery in the trial population.

The group statistics of primary and secondary outcomes are presented in Table 2 and Supplementary Table S1. On Day 7, 62/120 (51.67%) patients in the Arbidol group and 71/116 (61.21%) patients in the favipiravir group (*p* = 0.1396) clinically recovered (difference in recovery rate (DRR): 0.0954, 95% confidence interval (CI): −0.0305∼0.2213) (Figure 2). Hence, we conclude that favipiravir does not show superior efficacy compared to Arbidol to improve the clinical recovery rate at day 7.

**TABLE 2:** Comparison of clinical recovery rate at Day 7.

Variables	Favipiravir group	Arbidol group	Rate ratio (95% CI)	*p* value
Total patients	(*n* = 116)	(*n* = 120)		0.1396
Recovered, *n* (%)	71 (61.21)	62 (51.67)	0.0954 (−0.0305, 0.2213)	
Moderate patients	(*n* = 98)	(*n* = 111)		
Recovered, *n* (%)	70 (71.43)	62 (55.86)	0.1557 (0.0271, 0.2843)	0.0199
Severe or critical patients	(*n* = 18)	(*n* = 9)		
Recovered, *n* (%)	1 (5.56)	0 (0.00)	0.0556 (−0.0503, 0.1614)	0.4712
Patients with hypertension and/or diabetes	(*n* = 42)	(*n* = 35)		
Recovered, *n* (%)	23 (54.76)	18 (51.43)	0.0333 (−0.1904, 0.2571)	0.7704

A post hoc test for the interaction between treatment and clinical classification showed no interaction between these two factors, both of which contributed to the primary outcome (*p* = 0.017 for treatment and *p* < 0.001 for clinical classification, with a general linear model). A post hoc analysis found that for moderate patients with COVID-19, the clinical recovery at day 7 was 62/111 (55.86%) in the Arbidol group and 70/98 (71.43%) in the favipiravir group (*p* = 0.0199) (DRR: 0.1557, 95% CI: 0.0271∼0.2843); for severe/critical patients, the clinical recovery rate was 0/9 (0%) in the Arbidol group and 1/18 (5.56%) in the favipiravir group (*p* = 0.4712) (DRR: 0.0556, 95% CI: −0.0503∼0.1614).

### Comparison of the Duration of Pyrexia, Cough Relief Time and Auxiliary Oxygen Therapy or Noninvasive Mechanical Ventilation Rate

**FIGURE 3:**
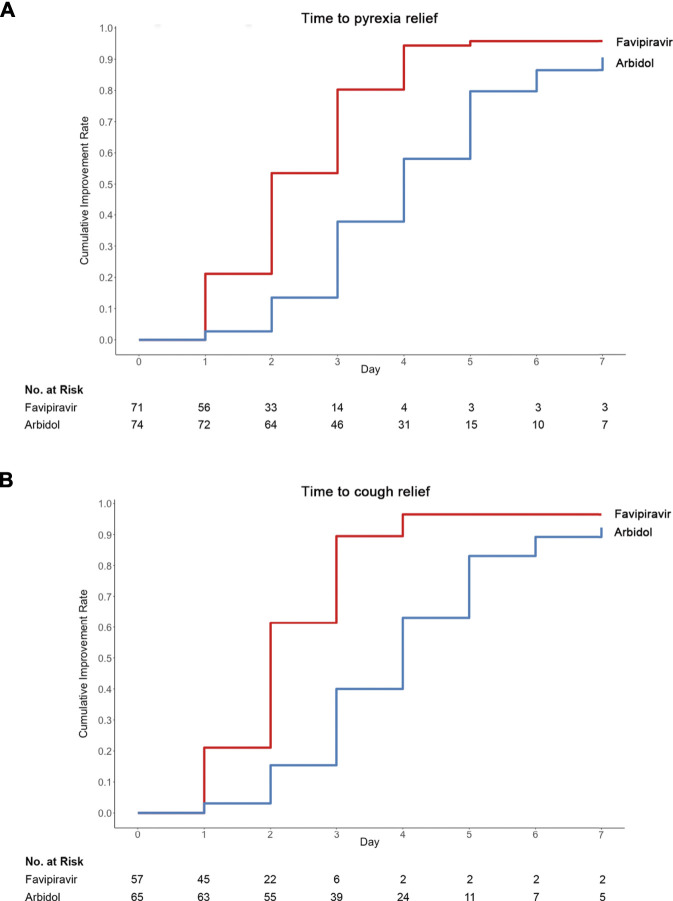
Time to **(A)** pyrexia or **(B)** cough relief in the trial population.

Table 3 displays the duration of pyrexia, cough relief time and auxiliary oxygen therapy or noninvasive mechanical ventilation rate between the favipiravir and Arbidol groups. At baseline, for patients in the favipiravir group, 71/116 (61.2%) had pyrexia and 78/116 (67.2%) had cough; for patients in the Arbidol group, 74/120 (61.7%) had pyrexia and 73/120 (60.8%) had cough. While the incidence of pyrexia and cough did not differ between the two groups at baseline, both the latency to pyrexia reduction and cough relief in the favipiravir group were significantly shorter than for those in the Arbidol group (*p* < 0.0001, Figure 3).

**TABLE 3:** Comparison of time to relief for pyrexia, cough relief time and other secondary outcomes.

Variables	Time to relief for pyrexia		Cough relief time	
	Favipiravir group	Arbidol group	Favipiravir group	Arbidol group
Total patients	(*n* = 71)	(*n* = 74)	(*n* = 78)	(*n* = 73)
Day 1	15 (21.13)	2 (2.70)	1 (1.28)	3 (4.11)
Day 2	23 (32.39)	8 (10.81)	2 (2.56)	1 (1.37)
Day 3	19 (26.76)	18 (24.32)	23 (29.49)	7 (9.59)
Day 4	10 (14.08)	15 (20.27)	20 (25.64)	11 (15.07)
Day 5	1 (1.41)	16 (21.62)	10 (12.82)	12 (16.44)
Day 6	—	5 (6.76)	10 (12.82)	10 (13.70)
Day 7	—	3 (4.05)	3 (3.85)	3 (4.11)
Day 8	—	—	7 (8.97)	6 (8.22)
Day 9	—	—	1 (1.28)	3 (4.11)
Censored	—	—	1 (1.28)	17 (23.29)
Log-rank *p* value	<0.0001		<0.0001	
**Other secondary outcomes**				
AOT or NMV/MV^a^	Favipiravir group	Arbidol group	Rate ratio (95% CI)	*p* value
Total patients	*n* = 116	*n* = 120		
With auxiliary, *n* (%)	21 (18.10)	27 (22.50)	−0.0440 (−0.1464, −0.0585)	0.4015
Patients with hypertension and/or diabetes	*n* = 42	*n* = 35		
With auxiliary, *n* (%)	9 (21.43)	10 (28.57)	−0.0714 (−0.2658, 0.1230)	0.4691
All-cause mortality	0 (0.00)	0 (0.00)	—	—
Dyspnea after taking medicine, *n* (%)	4 (3.45)	14 (11.67)	—	0.0174
Respiratory failure, *n* (%)	1 (0.86)	4 (3.33)	—	0.3700^a^
ICU admission	2 (1.72)	2 (1.67)	—	1.0000^a^

a Fisher’s exact test was used for comparison between groups.

**Abbreviations:** AOT: Auxiliary oxygen therapy; NMV: Noninvasive mechanical ventilation; MV: Mechanical ventilation.

The incidence of *de novo* AOT or NMV/MV was 27/120 (22.50%) in the Arbidol group and 21/116 (18.10%) in the favipiravir group (*p* = 0.4015) (DRR: −4.40%, 95% CI: −14.64∼5.85%). For all cases enrolled in this study, the all-cause mortality was 0. The number of cases of respiratory failure was 4 in the Arbidol group and 1 in the favipiravir group (*p* = 0.3700). Patients with dyspnea were 15/120 (12.5%) in the Arbidol group and 13/116 (11.2%) in the favipiravir group (*p* = 0.7588). A post hoc analysis showed that *de novo* incidences of dyspnea during the course of treatment occurred in 4/116 (3.45%) patients in the favipiravir group and 14/120 (11.67%) patients in the Arbidol group (*p* = 0.0174). Hence, we conclude that for secondary outcomes, favipiravir significantly shortened the latency to relieve cough and pyrexia. Four patients with severe COVID-19 (2 in the favipiravir group and 2 in the Arbidol group) had acute respiratory failure, septic shock, tachycardia, and transient hypotension; they received invasive mechanical ventilation and were admitted to the intensive care unit (Table 3). Of these patients, three had completed the 7 day medication regimen and only one discontinued the study drug because of adverse effects.

### Comparison of Antiviral-Associated Adverse Effects

During this trial, we detected 37 incidences of antiviral-associated adverse effects (AEs) in the favipiravir group and 28 incidences in the Arbidol group. All observed AE incidences were level 1. Favipiravir was associated with increased serum uric acid for 3 (2.50%) patients in the Arbidol group vs. 16 (13.79%) patients in the favipiravir group, *p* = 0.0014). No significant difference was observed for the frequency of abnormal ALT/AST, psychiatric symptom reactions or digestive tract reactions (Table 4). Most of these adverse reactions disappeared by the time the patients were discharged.

**TABLE 4:** Comparison of antiviral-associated adverse effects.

Adverse effects	Favipiravir group (*n* = 116)		Arbidol group (*n* = 120)		*p* value
	Frequency	Cases, *n* (%)	Frequency	Cases, *n* (%)	
Total	43	37 (31.90)	33	28 (23.33)	0.1410
Abnormal LFT	10	10 (8.62)	12	12 (10.00)	0.7156
Raised serum uric acid	16	16 (13.79)	3	3 (2.50)	0.0014
Psychiatric symptom reactions	5	5 (4.31)	1	1 (0.83)	0.1149^a^
Digestive tract reactions	16	16 (13.79)	17	14 (11.67)	0.6239

a Fisher’s exact test was used for comparison between groups.

## Discussion

We conducted a prospective, multicenter, open-label, randomized superiority inpatient clinical trial. This randomized trial found that favipiravir is not superior to Arbidol in terms of clinical recovery, but that favipiravir is superior in efficacy for the relief of moderate symptoms and accelerates the clinical recovery of pyrexia and cough compared with Arbidol. Only two severe patients progressed to respiratory failure in the favipiravir group and was put into the ICU.

The dosage amount of favipiravir in the phase 3 trials for adult influenza in the United States is a loading dose of 1800 mg twice a day on day 1 and a maintenance dose of 800 mg twice a day on days 2–5 (Du and Chen, 2020). A favipiravir trial in adults for Ebola virus disease is planned with three doses of 2400 mg/2400 mg/1,200 mg every 8 h on Day 1 and a maintenance dose of 1,200 mg twice a day afterward (Mentre et al., 2015). For treating influenza, the drug instruction recommended for favipiravir is 1600 mg/600 mg BID for 5 days, according to the pharmacokinetics, pharmacodynamics and IC50 properties of the drug. All participants in the favipiravir arm of this study received a favipiravir dosing regimen of 1600 mg/600 mg BID.

The aim of this study was to evaluate the improvement of the clinical recovery rate at day 7 with favipiravir for most moderate COVID-19 patients. The most common symptoms of COVID-19 were fever, cough, nasal obstruction, myalgia, gustatory and olfactory dysfunction, and sore throat (Guan et al., 2020). Clinical recovery was defined as continuous (>72 h) recovery of body temperature, respiratory rate, blood oxygen level and cough relief after treatment. The clinical recovery rate at day 7 of drug administration is critical to the progression and outcome of pneumonia. The endpoint, hence, is chosen based on clinical relevance.

On average, patients were enrolled in the study earlier in their disease course (a median of 9 days in the favipiravir/Arbidol group). At the time of this clinical trial initiation, clinical symptoms other than molecular diagnosis were used to confirm COVID-19 in China (Feb. to Mar. 2020). Following the clinical guidelines, we confirmed that all our patients had characteristic findings on CT imaging, including multiple, patchy, ground-glass opacity, subpleural linear abnormality, cobblestone pattern, or consolidation shadows, mainly distributed in the peripheral and subpleural areas of both lungs. Considering the population prevalence of COVID-19 at the time in Wuhan, we consider these criteria suitable to confirm COVID-19 patients.

In this trial, the favipiravir treatment did not improve the clinical recovery rate at day 7 (61.21%) compared to the Arbidol group (51.67%). This may be due to insensitivity to detect the difference due to having low doses and starting dosing late for patients, especially those with severe COVID-19. However, it did significantly improve the latency to cough relief and decreased the duration of pyrexia.

Four patients (2 in the favipiravir group and 2 in the Arbidol group) had acute respiratory failure with septic shock, tachycardia, and transient hypotension; they received invasive mechanical ventilation and were admitted to the intensive care unit (ICU). Three of these patients finished a 7 days dosing regimen, and only one discontinued the study drug. Thus, most of the patients completed the 7 days medication regimen. Favipiravir was not associated with any differences in ICU admission, AOT/NMV/MV, dyspnea, respiratory failure or all-cause mortality.

Interestingly, post hoc observation showed that favipiravir was effective in improving the clinical recovery rate at day 7 of drug administration in moderate COVID-19 patients compared to Arbidol. However, this effect diminished for severe/critical COVID-19 patients. Additionally, post hoc analysis showed that for moderate COVID-19 patients, favipiravir was associated with decreased auxiliary oxygen therapy or noninvasive mechanical ventilation rate with marginal significance (*p* = 0.0541). Finally, in the FAS, post hoc analysis also showed that favipiravir treatment significantly decreased *de novo* incidences of dyspnea. Whether favipiravir would be effective only for moderate COVID-19 patients or could be used to prevent disease progression is a question that warrants future investigation.

The combination of traditional Chinese medicine and antiviral drugs is more common in China, which is due to the traditional medical culture background of the treatment of choice. Additionally, anti-infection and immune regulation play an important role in the treatment of COVID-19. Ancillary treatments, such as traditional Chinese medicine, anti-infection and immunomodulatory drugs, were not significantly different between the groups (Supplementary Table S2).

Favipiravir was associated with increased serum uric acid but not abnormal ALT/AST, psychiatric symptom reactions or digestive tract reactions. Routine 12-lead electrocardiogram (ECG) measurements were measured routinely at baseline and discharge. We did not observe this phenomenon in the detection of 12-lead ECG when patients were being discharged. Abnormal ECG was reported in 34 of 116 patients in the favipiravir group, whereas only five patients had normal baseline levels and abnormal examinations at discharge. The most common adverse effects were mild changes in the ST segment, sinus arrhythmia, and sinus bradycardia with irregular law. Moreover, most of these adverse reactions disappeared by the time patients were discharged. Thus, the results suggest an unrelated relationship between abnormal ECG and favipiravir treatment. Therefore, we conclude that the dosing regimen for favipiravir in this study is safe for patients.

Considering our real-world experience at that time, hospitalized patients were of moderate (>80%), severe (10–20%) and critical types of COVID-19. The enrolled patients were not pre-selected by severity. For most moderate patients, it was relatively uncommon for them to deteriorate into states requiring respiratory support, including AOT or NMV/MV. Hence, most patients recovered without respiratory deterioration. Expecting a lower incidence of respiratory deterioration, we consider that we should need a larger number of patients with a limited timeframe to achieve similar statistical power. In fact, data from this study show that the incidence of *de novo* AOT or NMV/MV was 27/120 (22.50%) in the Arbidol group and 21/116 (18.10%) in the favipiravir group. Only one severe-type patient progressed to a critical state and was put into the ICU. This study was conducted in a demanding clinical research environment, and the observation time frame was limited due to the urgency of this epidemic. It is necessary to select a reasonable primary endpoint that maximizes the degree of efficacy differentiation to shorten the overall clinical study time and reduce the sample size as much as possible. We found that the duration of hospital stay for most patients was 7–15 days after enrollment from follow-up records. Therefore, the 7 days clinical recovery rate may be more suitable for the endpoint setting. Our trial has several limitations. First, for COVID-19, there is no clinically proven effective antiviral drug to serve as the control arm. Although the updated Chinese guidelines have recommended several options, including Arbidol (Furuta et al., 2002), no randomized clinical trial results on these drugs have been reported. Arbidol was widely used by Chinese doctors in the beginning stage of this epidemic of COVID-19 (Jan. 1–30, 2020) based on *in vitro* evidence (Li et al., 2020). For ethical reasons, we chose Arbidol for the control arm. Second, the observation time frame was limited due to the urgency of this epidemic. For the same reason, no relapse (including nucleic acid conversion, pyrexia, cough, or pneumonia progression by radiology) tracking was performed for the discharged patients. Third, all first-listed diagnoses of hospitalized COVID-19 patients present positive qRT-PCR on admission. However, 46.55% of patients in the favipiravir group and 38.33% in the Arbidol group were nucleic acid positive before taking the drugs. The accuracy of nucleic acid assays was limited, including previous treatment, latency of onset, sampling method, and biological specimen characteristics, which might cause the nucleic acid tests of some subjects to turn negative. This particular accuracy problem is a known issue among clinical practitioners worldwide. It was estimated that the assay might have at most 30–50% sensitivity for patients in the early stage of the disease, while contact history, clinical manifestations, radiology evidence, and laboratory results, including leukopenia and lymphopenia, could be confirmatory for these nucleic acid-negative pneumonia patients. Fourth, the protocol does not prespecify clinical classification and age as a stratification factor. Although there was no significant difference in the proportion of elderly patients between the two groups, 41 (34.17%) in the Arbidol group vs. 29 (25.00%) in the favipiravir group were ≥65 years old. Age still may produce a bias in the results. Ethical concerns arose against completely excluding severe/critical cases from potential beneficial treatment. Additionally, because of the complexity of the disease, progression from moderate to severe/critical is possible. Terminating trial treatment for such patients from the study was considered unacceptable. Post hoc analysis showed that both treatment and clinical classification contributed significantly to the primary outcome of the clinical recovery rate at day 7. The difference in the frequency of severe/critical patients between the groups reached marginal significance, which impacted the trial outcome. Fifth, there was no efficacy difference between favipiravir and Arbidol in improving the clinical recovery rate at day 7 of drug administration, which could be due to the choice of drug dose or the time to start taking medication.

## Conclusion

Compared to Arbidol, favipiravir does not significantly improve the clinical recovery rate at day 7. Favipiravir is associated with significantly shortened latency to relief for pyrexia and cough. The antiviral-associated adverse effects of favipiravir are mild and manageable.

## Data Availability

The original contributions presented in the study are included in the article/Supplementary Material, further inquiries can be directed to the corresponding author.
