# Supplementary material for: Favipiravir Versus Arbidol for Clinical Recovery Rate in Moderate and Severe Adult COVID-19 Patients: A Prospective, Multicenter, Open-Label, Randomized Controlled Clinical Trial
Source: Front Pharmacol. 2021 Sep 2;12:683296. doi: 10.3389/fphar.2021.683296 (PMC8443786; doi:10.3389/fphar.2021.683296)
Supplement: Supplementary file 1 [file DataSheet1.PDF]

Supplemental Material for

Favipiravir versus Arbidol for improving clinical recovery rate and syndromic alleviation in moderate and severe adult COVID-19 patients: A prospective, multicenter, open-label, randomized controlled clinical trial

Chang Chen[#], Yi Zhang[#], Jianying Huang[#], Ping Yin[#], Zhenshun Cheng, Jianyuan Wu, Song Chen, Yongxi Zhang, Bo Chen, Mengxin Lu, Yongwen Luo, Lingao Ju, Jingyi Zhang, Xinghuan Wang[*]

Table of Contents

Supplementary Tables 2

Supplementary Table S1. Group statistics of primary and secondary outcomes. 2

Supplementary Table S1-1. Comparison of clinical recovery rate of Day 7. 2

Supplementary Table S1-2. Comparison of duration of fever. 3

Supplementary Table S1-3. Comparison of duration of cough relief time. 4

Supplementary Table S1-4. Comparison of AOT or NMV. 5

Supplementary Table S1-5. Comparison of other secondary outcomes. 6

Supplementary Table S2. Comparison of the COVID-19 patients in combination with the drugs. 7

Supplementary Figures 8

Supplementary Figure S1. Categorial and continuous clinical feature distribution of all FAS patients. 8

Supplementary Figure S2. Swimmer's plot of clinical manifestion for all FAS patients. 10

Supplementary Figure S3. Ancillary drug usage for all FAS patients. 12

Supplementary Tables

Supplementary Table S1. Group statistics of primary and secondary outcomes.

Supplementary Table S1-1. Comparison of clinical recovery rate of Day 7.

Variables	FAS		PPS	
	Favipiravir group	Arbidol group	Favipiravir group	Arbidol group
**Total patients**	**n = 116**	**n = 120**	**n = 116**	**n = 115**
**Recovered, n (%)**	71(61.21)	62 (51.67)	71 (61.21)	58 (50.43)
**P value**	0.1396		0.0992	
**Rate ratio (95% CI)**	0.0954 (-0.0305, 0.2213)		0.1077 (-0.0196, 0.2351)	
**Moderate patients**	**n = 98**	**n = 111**	**n = 98**	**n = 111**
**Recovered, n (%)**	70 (71.43)	62 (55.86)	70 (71.43)	58 (54.72)
**P value**	0.0199		0.0136	
**Rate ratio (95% CI)**	0.1557 (0.0271, 0.2843)		0.1671 (0.0368, 0.2974)	
**Severe or critical patients**	**n = 18**	**n = 9**	**n = 18**	**n = 9**
**Recovered, n (%)**	1 (5.56)	0 (0.00)	1 (5.56)	0 (0.00)
**P value**	0.4712		0.4712	
**Rate ratio (95% CI)**	0.0556 (-0.0503, 0.1614)		0.0556 (-0.0503, 0.1614)	

Supplementary Table S1-2. Comparison of duration of fever.

Time	FAS		PPS	
	Favipiravir	Arbidol	Favipiravir	Arbidol
**Moderate patients**	**(n = 57)**	**(n = 65)**	**(n = 57)**	**(n = 62)**
Day 1	12 (21.05)	2 (3.08)	12 (21.05)	2 (3.23)
Day 2	23 (40.35)	8 (12.31)	23 (40.35)	8 (12.90)
Day 3	16 (28.07)	16 (24.62)	16 (28.07)	13 (20.97)
Day 4	4 (7.02)	15 (23.08)	4 (7.02)	15 (24.19)
Day 5	0 (0.00)	13 (20.00)	0 (0.00)	13 (20.97)
Day 6	0 (0.00)	4(6.15)	0 (0.00)	4 (6.45)
Day 7	0 (0.00)	2(3.08)	0 (0.00)	2 (3.23)
Day 8	-	-	-	-
Day 9	-	-	-	-
Censored	2 (3.51)	5(7.69)	2 (3.51)	5 (8.06)
**Log-rank P value**	< 0.0001		< 0.0001	

Supplementary Table S1-3. Comparison of duration of cough relief time.

Time	FAS		PPS	
	**Favipiravir**	**Arbidol**	**Favipiravir**	**Arbidol**
**Moderate patients**	**n = 60**	**n = 64**	**n = 60**	**n = 62**
Day 1	1 (1.67)	3 (4.69)	1 (1.67)	3 (4.84)
Day 2	1 (1.67)	1 (1.56)	1 (1.67)	1 (1.61)
Day 3	21 (35.00)	7 (10.94)	21 (35.00)	7 (11.29)
Day 4	18 (30.00)	11 (17.19)	18 (30.00)	11 (17.74)
Day 5	9 (15.00)	12 (18.75)	9 (15.00)	11 (17.74)
Day 6	7 (11.67)	10 (15.63)	7 (11.67)	10 (16.13)
Day 7	2 (3.33)	3 (4.69)	2 (3.33)	3 (4.84)
Day 8	1 (1.67)	4 (6.25)	1 (1.67)	4 (6.45)
Day 9	0 (0.00)	1 (1.56)	0 (0.00)	1 (1.61)
Censored	0 (0.00)	12 (18.75)	0 (0.00)	11 (17.74)
**Log-rank P value**	< 0.0001		< 0.0001	

Supplementary Table S1-4. Comparison of AOT or NMV/MV.

Variables	FAS		PPS	
	Favipiravir	Arbidol	Favipiravir	Arbidol
**Total patients**	**n = 116**	**n = 120**	**n = 116**	**n = 115**
**AOT or NMV/MV, n (%)**	21 (18.10)	27 (22.50)	21 (18.10)	26 (22.61)
**P value**	0.4015		0.3951	
**Rate ratio (95% CI)**	-0.0440 (-0.1464, 0.0585)		-0.0451(-0.1488, 0.0587)	
**Moderate patients**	**n = 98**	**n = 111**	**n = 98**	**n = 111**
**AOT or NMV, n (%)**	8 (8.16)	19 (17.12)	8 (8.16)	18 (16.98)
**P value**	0.0541		0.0592	
**Rate ratio (95% CI)**	-0.0895 (-0.1781, -0.0009)		-0.0882 (-0.1779, 0.0015)	
**Severe or critical patients**	**n = 18**	**n = 9**	**n = 18**	**n = 9**
**MV, n (%)**	13 (72.22)	8 (88.89)	13 (72.22)	8 (88.89)
**P value**	0.3261		0.3261	
**Rate ratio (95% CI)**	-0.1667 (-0.4582, 0.1248)		-0.1667 (-0.4582, 0.1248)	
**Patients with hypertension and/or diabetes**	**n = 43**	**n = 35**	**n = 42**	**n = 33**
**NMV/MV, n (%)**	9 (21.43)	10 (28.57)	9 (21.43)	10 (30.30)
**P value**	0.4691		0.3804	
**Rate ratio (95% CI)**	-0.0714 (-0.2658, 0.1230)		-0.0887 (-0.2887, 0.1112)	

AOT: Auxiliary oxygen therapy.
NMV: Noninvasive mechanical ventilation.
MV: Mechanical ventilation.

Supplementary Table S1-5. Comparison of other secondary outcomes.

Variables	Favipiravir group (n = 116)		Arbidol group (n = 120)
**All cause mortality, n (%)**	0 (0.00)		0 (0.00)
Statistics		/	
P value		/	
**Respiratory failure, n (%)**	1 (0.86)		4 (3.33)
Statistics		-	
P value		0.3700*	
**Dyspnea, n (%)**	13 (11.21)		15 (12.50)
Statistics		0.0943	
P value		0.7588	
**Entering ICU, n (%)**	2 (1.72)		2 (1.67)
Statistics		-	
P value		1.0000*	

*Fisher's exact test was used for comparison between groups.

Supplementary Table S2. Comparison of the COVID-19 patients in combination with the drugs.

Combined drugs[#]	Favipiravir group	Arbidol group	P value
**Moderate patients**	n = 98	n = 111	
Total	81 (82.65)	93 (83.78)	0.8271
Antibiotic	21 (21.43)	20 (18.02)	0.5355
Antiviral drugs	8 (8.16)	25 (22.52)	0.0045
Glucocorticoid	2 (2.04)	8 (7.21)	0.1078
Chinese herbal medicine	74 (75.51)	85 (76.58)	0.8569
Psychotropic substances	10 (10.20)	22 (19.82)	0.0540
Immunomodulator	5 (5.10)	15 (13.51)	0.0391
Nutritional support	17 (17.35)	11 (9.91)	0.1152
**Severe or critical patients**	n = 18	n = 9	
Total	17 (94.44)	8 (88.89)	1.0000*
Antibiotic	13 (72.22)	3 (33.33)	1.0000*
Antiviral drugs	3 (16.67)	2 (22.22)	1.0000*
Glucocorticoid	3 (16.67)	2 (22.22)	1.0000*
Chinese herbal medicine	15 (83.33)	7 (77.78)	1.0000*
Psychotropic substances	4 (22.22)	4 (44.44)	0.3748
Immunomodulator	3 (16.67)	1 (11.11)	1.0000*
Nutritional support	3 (16.67)	1 (11.11)	1.0000*

*Fisher's exact test was used for comparison between groups.
#Combined drugs:
Antibiotic: Moxifloxacin Hydrochloride Tablets, Cephalosporins
Antiviral drugs: Ribavirin Injection, Chloroquine Phosphate, Recombinant Human Interferon
Chinese herbal medicine: Lianhua Qingwen Capsule, Qiangli Pipa Lu, Xuebijing Injection
Psychotropic substances: Estazolam Tablets, Alprazolam Tablets, Dexmedetomidine, Diazepam
Immunity enhancing drugs: Human Serum Albumin (HSA), Thymalfasin
Nutritional support: Intacted Protein Enteral Nutrition, Vitamin C/B6/B12/Multivitamin

Supplementary Figures

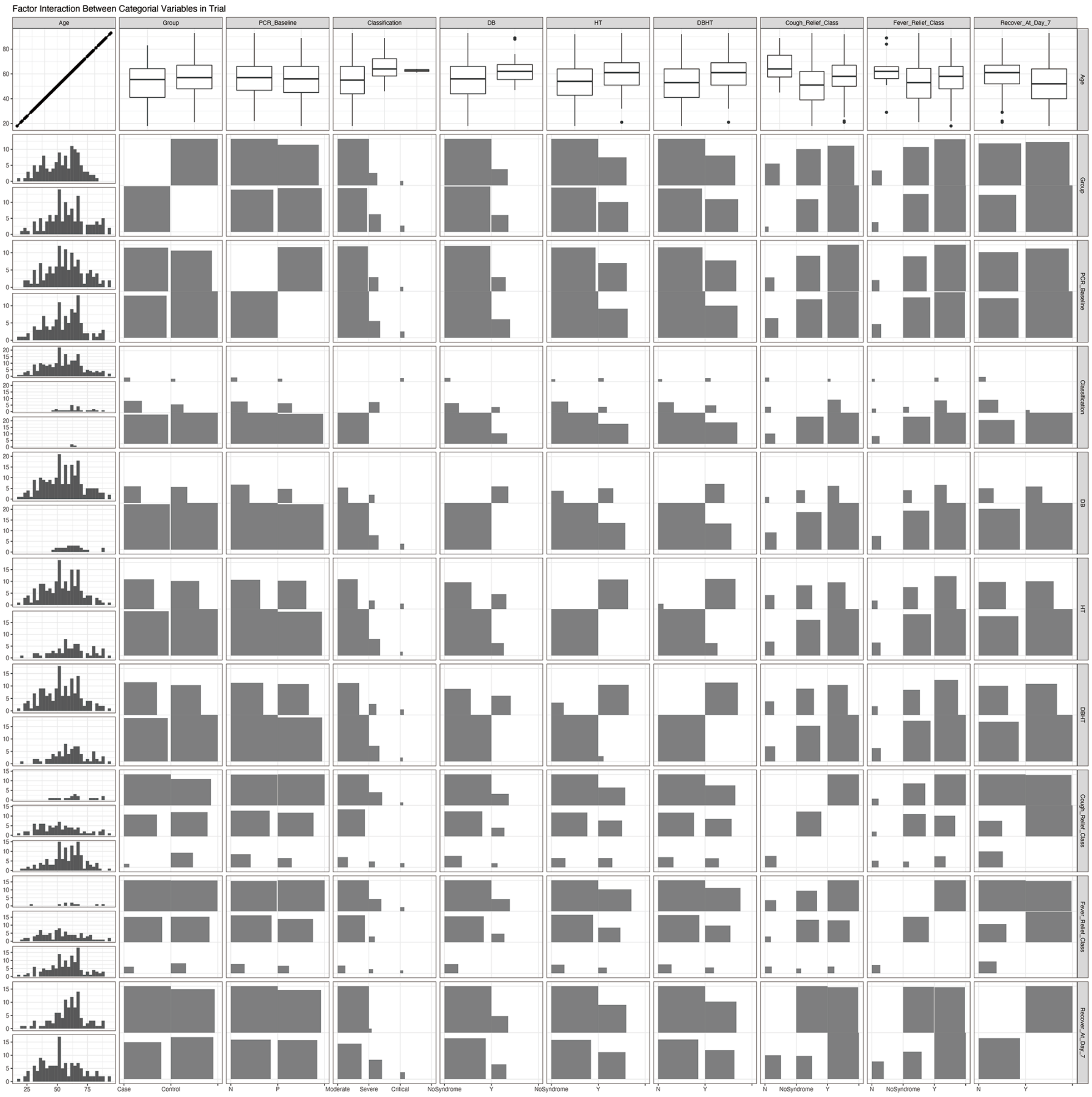

Supplementary Figure S1. Categorial and continuous clinical feature distribution of all FAS patients.

X and Y axis of this combinatorial figure was collected clinical features for all FAS patients. For continuous variable x categorial variable combinations, the distribution histogram of individuals was shown for each categorial variable. For categorial variable x continuous variable combinations, a boxplot was shown for the continous variable in groups of categories. For categorial variable x categorial variable

combinations, square area denotes the number of patients with such combination. Feature names: Group: case is the favipiravir group and control is the arbidol group. PCR baseline: N is negative and P is positive for SARS-CoV-2. Classification: clinical classification of moderate, severe and critical patients. DB: diabetes. HT: hypertension. DBHT: diabetes and/or hypertension. Cough Relief Class: Y is relieved, N is not relieved, and NoSyndrome means not coughing at the time of enrollment. Fever Relief Class: Y is relieved, N is not relieved, and NoSyndrome means no fever at the time of enrollment. Recovery at Day 7: the primary outcome, where Y is positive and N is negative. Analysis and plot were done by GGally (1.4.0) package in R (3.6.2).

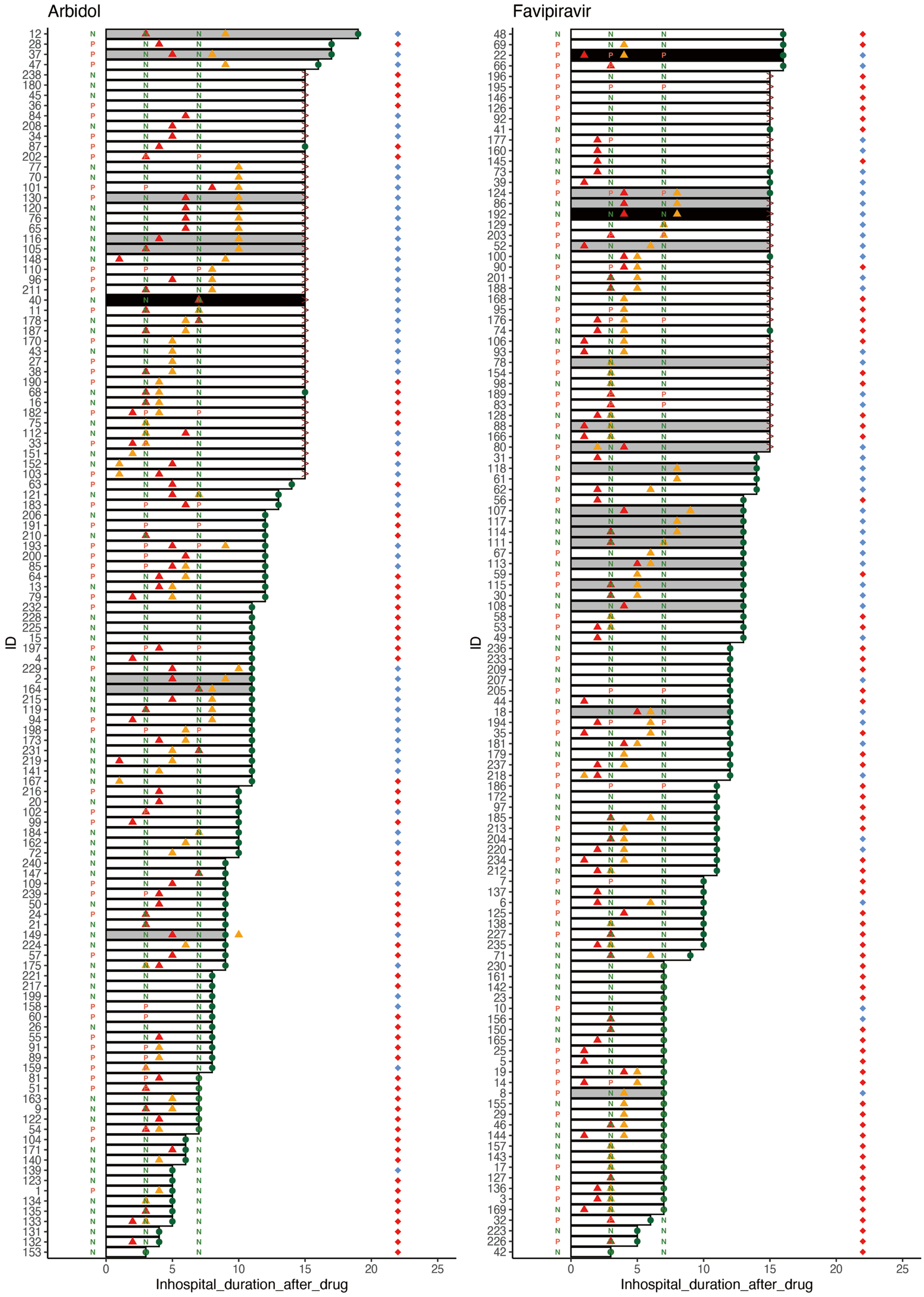

Supplementary Figure S2. Swimmer's plot of clinical manifestion for all FAS patients.

X axis denotes the days beginning from first dosing, and patients were distributed on Y axis. Each lane denotes a patient, where the swimming lane length denotes inhospital observation time. Clinical classification of each patient was colored in the lane as white (moderate), gray (severe) or black (critical). Discharge events were denoted as green circle (usually at the end of lane). Continuous inhospitalization were denoted as red arrowhead at the end of lane. Fever relief events were denoted as red triangle. Cough relief events were denoted as yellow triangle. PCR tests for SARS-CoV-2 were performed at Day 0 (before enrollment, on the left of each lane), Day 3 and Day 7, where a red "P" denotes positive result and a green "N" denotes negative result. The primary endpoint, recovery of syndromes at Day 7, was denoted at the rightmost of the panel as red (recovered, a positive outcome) or blue (not recovered, a negative outcome) rhombics. Analysis and plot were done by R (3.6.2).

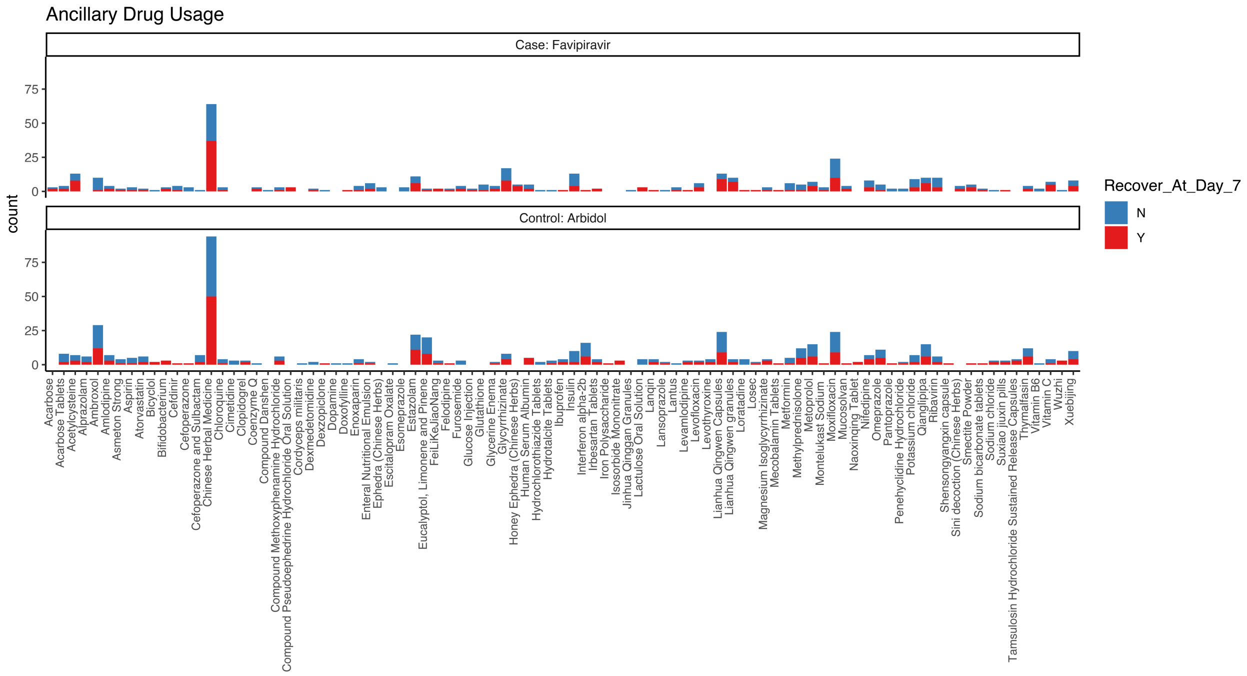

Supplementary Figure S3. Ancillary drug usage for all FAS patients.

X axis denotes drug used and Y axis denotes the number of patients using this drug. Column filling color denotes the primary outcome of patients: red is not recovered at Day 7 (negative result) and blue is recovered (positive result).
